# Teamwork and Decision Making among Basketball Referees: The 3PO Principle, Refereeing Level, and Experience

**DOI:** 10.5114/jhk/169439

**Published:** 2023-07-25

**Authors:** Eran Sabag, Ronnie Lidor, Michal Arnon, Elia Morgulev, Michael Bar-Eli

**Affiliations:** 1 Department of Movement and Sport Sciences, Levinsky-Wingate Academic College, Netanya, Israel.; 2 Department of Physical Education, Kaye Academic College of Education, Beer-Sheva, Israel.; 3 Department of Business Administration, Guilford Glazer Faculty of Business and Management, Ben-Gurion University of the Negev, Beer-Sheva, Israel.

**Keywords:** positioning, coordination, three-person officiating, accuracy of decisions

## Abstract

In this study, the three-person officiating (3PO) principle was employed as an innovative method to examine decision-making (DM) processes among basketball referees. We aimed at exploring whether the ranking, experience, and teamwork among 25 basketball referees could predict accuracy of DM in ambiguous situations taken from basketball games. An analysis of 283 officiating cases taken from 100 filmed games was conducted. The events were then classified by nine experts according to whether the officiating decision was accurate, and which referee (Lead, Centre or Trail) was standing in the main coverage area, as per the 3PO principle, when the decision was made. Our findings indicate that the teamwork (coordination) component was associated with the quality of DM. Of the 283 events, 60 decisions (21%) were not made from the recommended position according to the 3PO principle; 49 of those decisions were incorrect. The findings are discussed from both developmental and instructional perspectives.

## Introduction

Referees are key stakeholders in both individual and team sports. Their primary responsibility is the maintenance of safety and competitive fairness through enforcement of the rules (Bar-Eli et al., 2011; Hancock et al., 2021). Single refereeing decisions can have major impact on the outcome (Dosseville et al., 2013; Hossner et al., 2019; Raab et al., 2019), and accurate decision-making (DM) performance is considered to be one of the most important characteristics that a referee should possess (Bar-Eli et al., 2011; Nabli et al., 2019). Ample research has been conducted to identify sources of referees' bias, and to reveal how accuracy of officiating can be improved (Dohmen and Sauermann, 2016; Morgulev et al., 2018; Schweizer et al., 2013; Spencer et al., 2020). Contributing factors that have been found to assist referees in their DM processes include physical fitness (Helsen and Bultynck, 2004; Leicht, 2008), perceptual ability (Helsen and Bultynck, 2004; Kittel et al., 2019; Cobanoglu et al., 2021), mental ability (Anshel et al., 2014; Castillo et al., 2017), visual attention (Pietraszewski et al., 2014; Spitz et al., 2018), rules knowledge, and game management (Mascarenhas et al., 2002).

One factor that seems to be particularly relevant to referees' DM quality is their level of expertise (MacMahon et al., 2007; Souchon et al., 2013). Expertise is the individual's ability to effectively perform in domain-specific tasks (Chassy and Gobet, 2010), and refers to the characteristics, skills, and knowledge that distinguish experts from novices or less experienced individuals (Ericsson et al., 2018; Ericsson and Pool, 2016). In sport, the referees' level of expertise is typically reflected by the level at which they officiate (e.g., regional, national or international levels) (Avugos et al., 2021).

DM of expert referees is superior to that of novice referees (Larkin et al., 2011; Put et al., 2013). For example, Gilis and colleagues (2008) found that international-level assistant referees were more accurate in recalling the spatial positions of soccer players in complex offside situations, compared to their national-level counterparts. Page and Page (2010) examined the influence of the home advantage on officiating, based on an extensive sample of games from various English soccer games. Those authors concluded that on average, referees with a greater level of expertise were less responsive to social pressure than those with less experience and training.

In another ball game, i.e., basketball, Hack et al. (2009) demonstrated that compared to non-professionals, professional referees exhibited superior domain-specific attention mechanisms. This was evident through them ascribing greater importance to officiating tasks and using long-time experience to assess effectively game situations. Overall, previous findings suggest that officiating expertise is associated with an improved ability to select and process relevant, useful situational information (Ghasemi et al., 2009; Hancock and Ste-Marie, 2013; Pizzera et al., 2018).

Another factor that distinguishes between novice and expert referees is related to their teamwork, that is, coordination and positioning in relation to the event and to each other (Avugos et al., 2021; Samuel et al., 2020). For example, Mallo and colleagues (2012) showed that DM accuracy of referees and assistant referees in soccer was affected by either the distance or the angle from which they observed the game. When examining key factors that contribute to expert officiating performance in the National Rugby League, Morris and O'Connor (2017) found positioning and teamwork to be among the leading attributes in officiating excellence. De Oliveira et al. (2011), however, did not find significant associations between the referee's distance from a foul play and the accuracy of the call in Brazilian soccer. Hossner et al. (2019) analyzed viewing angles and distances in relation to error rates in whistled and non-whistled events in the International Federation of Association Football (FIFA) World Cup 2014. They also found no significant correlations between the variables, yet they stated that although there seemed to be no “ideal distance” for making accurate decisions in soccer, elite-level referees were able to effectively position themselves in relation to an anticipated event.

Indeed, referees' DM has been examined under a variety of conditions, including different officiating ranks, court locations, and distances from the given game situation. Yet referees' on-court coordination and collaborations during games, as well as their impact on the referees' shared DM in practice, have been largely neglected in sports refereeing literature. In other words, despite referees' DM requiring teamwork and cooperation, especially in ball games such as basketball, in-depth research on this topic is greatly lacking (Pina et al., 2021). Moreover, since the introduction of the three-person officiating (3PO) principle in basketball in the beginning of the 2000s, only few studies have examined the practical application of the referees' teamwork, regarding cases where referees changed positions during the actual game.

### The 3PO Principle

**Figure 1:**
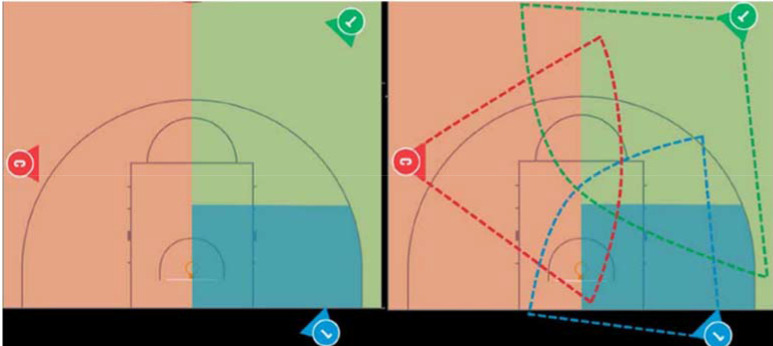
Lefthand panel: Theoretical 3PO coverage, Lead (Blue), Centre (Red), and Trail (Green); Righthand panel: Real-time, functional court coverage, Lead (Blue), Centre (Red), and Trail (Green).

In any professional game of basketball, three referees simultaneously function on the court, working together as a single unit by what is termed *the 3PO principle*. According to this notion, the referees alternate their zone of responsibility, as the location of the ball and the players changes in real time [for more details, see the International Basketball Association (FIBA) Three-Person Officiating Manual: https://www.clubdelarbitro.com/documentos/FIBA_3PO_Advanced_v1_1_Dec2020_en.pdf (accessed on 1 July 2022)]. Each referee fills one of three basic positions: Lead, Centre or Trail. Each position is responsible for a primary coverage area (Figure 1, lefthand panel). In some cases, due to functional coverage areas (Figure 1, righthand panel), some overlapping does occur between the three referees.

### Addressing the 3PO Principle for Increasing Validity of DM Studies on Basketball Officiating

The literature is abundant in studies on referee positioning in soccer (Avugos et al., 2021; Kittel et al., 2019), yet much less attention has been given to positioning and coordination among the triad of referees that is unique to the game of basketball (for a recent systematic review on basketball referees, see García-Santos et al., 2020). In a preliminary descriptive study, Smid (2015) attempted to characterize the positions and zones of responsibility among the three referees on the court (Lead, Centre, and Trail). It was found that over three basketball seasons, the Lead position was dominant compared to the Centre and Trail positions. Moreover, changes to the team's shared DM indicated a diffusion of power between the team members. Overall, the Lead position outperformed the other two positions, yet no effect of the officiating referees' DM on accuracy was found. The current study is novel as it focuses on the teamwork component of DM in basketball referees.

For educational purposes, Pecev et al. (2016) applied neural network techniques to simulate the visual field and identify the optimal relative positioning for each of the three referees in various game situations. Finally, Hrusa and Hrusova (2021) used expert observers to assess the accuracy of referees in court decisions. Comparing between 30 games that were officiated by two referees and 30 games that were attended by three referees (all from the same league), those authors concluded that the average number of officiating mistakes decreased significantly with the transition from a two- to a three-referee system.

In the current study, we applied a unique methodological approach to research on basketball officiating, by incorporating the quality of the referees' DM, their officiating level, years of experience, and the novel additional component of the 3PO principle. By adding this principle as a real-game variable, we strove to increase the ecological validity of how DM processes in basketball referees were assessed from a teamwork perspective.

We hypothesized that the manner in which referees in basketball coordinated their actions during an actual game – namely, how they applied the 3PO principle – would influence the quality of their DM as follows: (a) maintaining or not maintaining the designated area of responsibility of each referee would influence the quality of his officiating calls that he made throughout the game; (b) the amount of overlapping during the game between the designated coverages of the referees would influence the accuracy of the referees' calls; and (c) the distance of each referee in his designated area from the actual officiating event would influence the quality of DM. In addition, we examined referees' expertise from two perspectives, namely, (a) were they national or international?, and (b) how many years of experience did they have?

## Methods

### Participants

Nine Israeli elite-level basketball referees served as expert observers in this study. They were all males, aged 36–52 years (mean = 43.9), with an average of 26.3 years of experience. The expert observers were recruited from the Israeli Basketball Referees Association and were active members of FIBA. All nine observers held an international refereeing license and had experience in officiating international games, such as in the EuroLeague and the European Championships for national teams.

In line with protocols used in previous studies on referees in sport (Brand et al., 2006; Morgulev et al., 2014; Plessner and Betsch, 2001; Sabag et al., 2018), expert observers were asked to provide their professional opinion on a sample of real-game situations, while assessing the quality of the referees' DM in each situation. The study was approved by the Ethics Committee of the authors' affiliated academic institution and by the Ethical Committee of the Israeli Basketball Referees Association.

### Database

WSC Sports Technologies software [https://wsc-sports.com (accessed on 1 January 2019)], was used to download 100 games from the Israeli Super League from the 2016–17 season. We scanned these games and chose 321 events that seemed to be the most ambiguous, with the potential to elicit officiating errors. All selected events constituted a complex situation in which players, fans, broadcasters, and/or coaches were dissatisfied with the referees' calls or no-call decisions.

The events were selected by the first author of this article, a 42-year-old basketball coach with 20 years of coaching experience, including working at the highest level of competitive basketball in Israel (The Israeli Super League). The Israeli Super League is the highest competitive basketball division (i.e., Division 1) in Israel. This league is comprised of 12 professional clubs. Players practice on a daily basis and play between one and two games per week. Some clubs also participate in the European leagues (e.g., the Euroleague)]. To validate the selection of these events, one experienced professional coach (male; 40 years old with 19 years of coaching experience) and one experienced professional referee (male; 37 years old with 13 years of officiating experience) reassessed the selected events. They were asked to confirm that each event constituted an ambiguous on-court situation, with the potential to elicit officiating errors. In all events, the referees and their calls were edited (by the first author) out of the video recordings. As such, the expert observers did not know whether the referee had made a call or had ignored the situation. The events were then grouped into videoclips of 20 events each.

## Procedures

For each selected event, the first author documented the location of the event on the court, the position of the three referees (Lead, Centre, and Trail) on the court, the referee who made the call, and his decision. In events where the referees did not blow their whistle, the officiating decision was classified as “no-call”.

Each event was independently examined by two expert observers. After comparing their input, a third expert observer was required in cases where a lack of agreement was seen between the first two experts. Each expert observer sat in front of a computer screen in a quiet room. The session began with a brief introduction and a short training session, in which three trial events were viewed and discussed, to ensure the expert observer had understood the requirements of the observational task.

Each officiating event was presented to the experts in the following sequence: (a) an introductory slide presenting the number of the event, with arrows pointing to players involved in the event; (b) a videoclip of the event itself, without the referees' decision; (c) two slow-motion replays of the event; and (d) a final slide requesting the classification of the event. For each officiating event, expert observers were asked to identify: (a) the referee that should have taken responsibility for the call when an event occurred within his primary coverage area; and (b) the correct officiating decision that should have been made. Each expert observer assessed 80–100 events.

For 38 of the 321 selected events (about 12%), a consensus was not reached between the three expert observers regarding the correct officiating decision and the referee who should have been responsible for the given event. These 38 unidentified cases were therefore excluded from the data analysis. Of the remaining 283 officiating events, agreement was not reached between the first two observers for only six cases (about 2%), which required the involvement of a third expert observer.

### Referees' Age and Years of Experience

A total of 25 referees were involved in the 283 cases that were included in this study. Seven were licensed international-level referees. Their age and years of officiating experience are presented in Table 1. A *t*-test indicated that international-level referees were older (*t* = 1.96; *p* < 0.05, Cohen's *d* = 0.87) and more experienced (*t* = 3.22; *p* < 0.01, Cohen's *d* = 1.44) than their national-level counterparts.

**Table 1:** Referees' age and years of experience by the national/international level.

Referees' level (*N*)	Mean age (*SD*) (years)	Mean years of experience in Division 1 (*SD*)
National (*n* = 18)	38.6 (5.7)	6.7 (5.1)
International (*n* = 7)	43.4 (4.7)	14.3 (5.9)
Total = 25	40.0 (5.8)	8.8 (6.2)

### Statistical Analyses

Chi-square tests were performed to examine correlations between the referees' level of officiating, teamwork, and quality of decisions. A two-way ANOVA was conducted to determine whether the distance from the event was related to the referees' level, teamwork, and quality of decisions. To examine robustness, we employed a binomial logistic regression in which quality of decisions and teamwork served as the dependent variables. Cramér's V, partial eta-squared, and Cohen's *d* were used as effect sizes to match the relevant statistical test. Alpha was set at 0.05 for all statistical analyses. SPSS version 26 was used for data analyses.

## Results

### Comparison of Decisions Made by Real-Time Referees vs. Expert Observers

Distribution of referees' and expert observers' decisions in the 283 officiating events is presented in Table 2 (cross-tabulation).

**Table 2:** Cross-tabulation of the decisions made by the referees and expert observers.

Referees									
		Foul	Shooting Foul	Offensive Foul	Travelling	No Call	Flagrant	Out	Total
**Expert Observers**
	Foul	16	3	5	0	8	1	1	34
	Shooting Foul	1	12	2	1	13	1	0	30
	Offensive Foul	6	6	12	0	17	0	0	41
	Travelling	0	0	0	4	12	0	0	16
	No Call	25	46	14	3	61	1	0	151
	Flagrant	3	1	0	0	2	4	0	10
	Goaltending	0	0	0	0	1	0	0	1
	Flop	0	0	1	0	0	0	0	1
Total	51	68	34	8	114	7	1	283

**Note:** The officiating decisions in Table 2 were identified by either referees or expert observers, and categorized as follows:

*Foul* (referees and experts): Illegal personal contact with a player in offense not in the act of shooting;

*Shooting foul* (referees and experts): Illegal personal contact with a player in offense in the act of shooting;

*Offensive foul* (referees and experts): Illegal personal contact with a player in defence;

*Travelling* (referees and experts): A player's illegal movement while holding a live ball;

*No call* (referees and experts): Ignored the situation/no refereeing decision was required;

*Flagrant* (referees and experts): An unsportsmanlike foul, contact with an opponent, illegitimately attempting to directly play the ball within the spirit and intent of the rules;

*Out* (referees): A player with the ball is out-of-bounds/causes the ball to go out-of-bounds;

*Goaltending* (experts): Situation during a shot when a player touches the ball while it is completely above the level of the ring on its downward flight to the basket or after it has touched the backboard;

*Flop* (experts): Deceptive behaviour of a player who intentionally falls after little or no physical contact by an opposing player in order to receive a foul.

Overall, expert observers only reached the same decision as referees in 109 of the 283 events (38.5%), confirming that the selected officiating situations were indeed challenging. The highest agreement rates were found in decisions regarding flagrant fouls (4 out of 7; 57.1%) and on the no-calls (61 out of 114; 53.5%). On the other hand, only 12 of the 68 calls of a shooting foul (17.6%) were confirmed by experts.

### Referees' Level, Teamwork, and Distance between the Referee and the Actual Event

The number and the percentage of incorrect/correct calls made by referees are presented in Table 3 according to their officiating level (national/international). These results show that referees at the international level did not exhibit better DM than their national level counterparts (*χ^2^*_(1)_ = 0.18, *p* > 0.05, *Rc* = 0.25).

**Table 3:** The number and the percentage of incorrect/correct calls made by referees according to their officiating level (national/international).

	Incorrect	%	Correct	%	Total
National	116	62.40%	70	37.60%	186
International	58	59.80%	39	40.20%	97
Total	174	61.50%	109	38.50%	283

We also examined cases where the referee who should have made the call (as the event occurred within his primary coverage area) was indeed the one who did take responsibility for DM. Our findings show that these cases were less erroneous than cases where the wrong referee made the call (i.e., when the event was outside his primary coverage area). The number and the percentage of incorrect/correct calls made by referees in incorrect/correct positions are presented in Table 4.

**Table 4:** The number and the percentage of incorrect/correct calls made by referees in incorrect/correct positions.

	Incorrect	%	Correct	%	Total
Incorrect teamwork	49	81.70%	11	18.30%	60
Correct teamwork	125	56.10%	98	43.90%	223
Total	174	61.50%	109	38.50%	283

As seen in Table 4, referees made more errors (81.7%) when they made decisions regarding events that were outside their primary coverage area (*χ^2^*_(1)_ = 13.09, *p* < 0.01, *Rc* = 0.22). This finding is also true when examining each group of referees discretely: national level referees (*χ^2^*_(1)_ = 10.01, *p* < 0.001, *Rc* = 0.23) and international level referees (*χ^2^*_(1)_ = 3.68, *p* < 0.05, *Rc* = 0.20). Evidently, correct positioning among the three referees (i.e., teamwork) is an important officiating factor. We therefore also examined whether international level referees outperformed their national level colleagues in this regard. The number and the percentage of incorrect/correct positions by national/international level referees are presented in Table 5.

**Table 5:** The number and the percentage of incorrect/correct positions (teamwork) by national-/international level referees.

	Incorrect teamwork	%	Correct teamwork	%	Total
National	35	18.80%	151	81.20%	186
International	25	25.80%	72	74.20%	97
Total	60	21.20%	223	78.80%	283

Results in Table 5 show that no evidence was observed for associations between the level of referees and the quality of teamwork (*χ^2^*_(1)_ = 1.85, *p* > 0.05, Rc = 0.08). We therefore performed a two-way ANOVA to determine whether teamwork and the referee's level were associated with the distance between the referee and the event. In cases with correct teamwork, the referee was found to be situated closer to the event, compared to cases with erroneous teamwork (5.88 m and 7.27 m, respectively) [*F*_(1, 279)_ = 32.72, *p* < 0.01, *η^2^* = 0.11]. Moreover, international level referees were found to be located further away from the event, compared to their national level counterparts (6.96 m and 6.19 m, respectively) [*F*_(1, 279)_ = 10.08, *p* < 0.01, *η*^2^ = 0.04].

In light of the significant interaction between teamwork and the referees' level [*F*_(1)_ = 7.08, *p* < 0.01, *η*^2^ = 0.03], we examined cases with correct/erroneous teamwork separately. Our findings reveal that in cases with correct teamwork, international and national level referees were found to be standing at a similar distance from the event (5.94 m and 5.81 m, respectively) [*F*_(1, 221)_ = 0.34 , *p* > 0.05, *η^2^* = 0.002]. However, in cases where teamwork was erroneous, referees at the international level were found to be standing further away from the event compared to their national level colleagues (7.98 m and 6.56 m, respectively) [*F*_(1, 58)_ = 7.06, *p* < 0.01, η^2^ = 0.11]. This finding indicates that when international level referees were involved, their erroneous teamwork was related to their tendency to take responsibility for events that took place further away from them.

**Figure 2:**
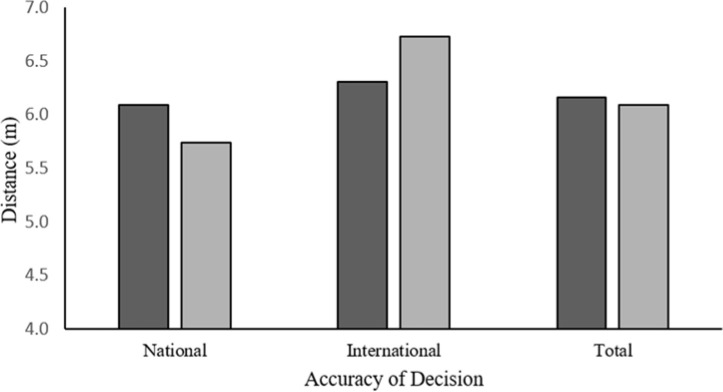
Distance between national and international level referees and the actual event in incorrect/correct decisions.

Analysis of accuracy rates of referees by the national/international level, and in relation to their distance from the given event, revealed that national level referees were at a similar distance from the event in both erroneous and correct decisions (6.09 m and 5.74 m, respectively) [*F*_(1, 184)_ = 2.00, *p* > 0.05, *η^2^* = 0.01]. International level referees were also found to be at a similar distance in both correct and erroneous decisions (6.72 m and 6.30 m, respectively) [*F*_(1, 95)_ = 1.22, *p* > 0.05, *η^2^* = 0.01]. Overall, international level referees made decisions from further away than their national level counterparts (6.47 m and 5.96 m, respectively) [*F*_(1, 281)_ = 5.6, *p* < 0.01, *η^2^* = 0.02], as seen in Figure 2. However, taking responsibility for events that occurred further away had no detrimental effect on their rate of accuracy. National level referees, on the other hand, needed to be closer to the event in order to make a correct decision.

### Robustness Check

To conduct a robustness check, a binomial logistic regression analysis was performed to assess the findings of this study. This was done through two models. Model 1 (Table 6) was used to assess the explanatory power of: (a) correct teamwork; (b) the referee's level; (c) referee's experience; and (d) the referee's distance from the event. Model 2 (Table 7) was used to assess the explanatory power of: (a) the referee's level; (b) referee's experience; and (c) the referee's distance from the event.

**Table 6:** Binomial Logistic Regression Analysis, Model 1.

							95% for	
							C.I EXP(B)	
	B	SE	Wald	Df	Sig.	Exp(B)	Lower	Upper
Teamwork	−1.35	0.38	12.76	1	0.00	0.25	0.12	0.54
Referee level	−0.21	0.31	0.45	1	0.50	0.81	0.43	1.49
Referee experience	−0.006	0.02	0.04	1	0.82	0.99	0.94	1.04
Referee distance	0.06	0.07	0.63	1	0.42	1.06	0.91	1.24

**Table 7:** Binomial Logistic Regression Analysis, Model 2.

							95% for	
							C.I EXP(B)	
	B	SE	Wald	Df	Sig.	Exp(B)	Lower	Upper
Referee level	−0.20	0.37	0.30	1	0.58	0.81	0.38	1.70
Referee experience	0.002	0.03	0.004	1	0.95	1.00	0.94	1.06
Referee distance	−0.44	0.09	21.85	1	0.001	0.64	0.53	0.77

Model 1 was found to be significant [*χ^2^* (4, *N* = 283) = 15.44, *p* < 0.001], explaining 7.2% (Nagelkerke *R^2^*) of the variance of the correct decisions and correctly classifying 60.8% of all observed situations. Model 2 was also found to be significant [*χ^2^* (3, *N* = 283) = 27.01, *p* < 0.001], explaining 14.1% (Nagelkerke *R^2^*) of the variance of the correct positions and correctly classifying 80.9% of the observed cases. As such, teamwork was the only significant predictor of accurate officiating (i.e., correct DM). Model 2 indicates that referees who made decisions from further away often hindered such events rather than contributed to correct DM, as these were outside their primary coverage area.

The results of binomial logistic regression analyses strengthened the findings of ANOVA analysis. Overall, findings indicate that national level referees needed to remain within the predefined boundaries of the 3PO principle, while higher degrees of freedom could be permitted among international level referees.

## Discussion

In this study, we examined the DM quality of basketball referees at the national and international level in ambiguous officiating situations, using an authentic real-game methodology, i.e., the 3PO principle. By innovatively applying this principle as a research variable, we attempted to explore relationships between the accuracy of the referees' officiating decisions, their officiating level, and their teamwork (based on the 3PO principle). Basketball situations that were examined in this study were derived from the Israeli Super League, i.e., the highest competitive basketball division in Israel.

Previous studies on basketball officiating found that employing the 3PO principle was better than employing the previous two-person officiating (2PO) principle in terms of the quality of referees' decisions (Hrusa and Hrusova, 2021). However, those earlier studies did not examine basketball referees' DM accuracy with reference to the designated position of each active referee according to the 3PO principle. In the present study, in order to assess DM accuracy, referees' decisions were compared to officiating decisions of the same situations made by expert observers in hindsight. We adopted this protocol in order to determine whether the referees who made the decisions were indeed standing in the position recommended by the 3PO principle, and whether their positions were associated with inaccurate/accurate calls. We hypothesized that maintaining the correct position in line with the 3PO principle would improve the referees' teamwork, and in turn, help them make more accurate on-court decisions.

Two main findings emerged from the current study. First, no associations were observed between the referees' ranking (national/international level), officiating experience, and DM accuracy. These findings differ from those of previous studies (Nevill et al., 2002; Pizzera and Raab, 2012; Spitz et al., 2021), where DM quality among referees from higher ranks and with more officiating experience was typically better than among their counterparts from lower level leagues and with less experience. The data collected in our study indicated that the quality of calls made by international level referees was not better than that of national level referees.

To explain these findings, we speculated that as both the national and the international level referees officiated at the same competitive level (i.e., the Israeli Super League, Division 1), rather than at different levels of competitive divisions/leagues, this minimized the gap between the two groups of referees. As such, we assumed that international level referees in each game assisted national level referees in their officiating performance, serving as on-court tutors, and providing the latter with on-court officiating support. In other words, the mix of referees from both the national and the international level upgraded officiating performances of national level referees.

The second main finding of our study was related to the implementation of the 3PO principle. The results revealed a significant association between the outcome of referees' DM and their positioning in the officiating situation: of the 283 analysed situations, expert observers identified 174 calls as incorrect (61.5%), of which 49 (28%) were associated with referees' incorrect teamwork as defined by the 3PO principle. No additional associations were found between any of the other DM variables examined and remaining incorrect officiating decisions (125; about 72%). In fact, 60 of the 283 officiating decisions (21%) were not made from the position recommended by the 3PO principle. As only 11 of them were correct (18%), the position within the 3PO principle of the referee seems to be a major factor determining DM success, regardless of the referees' ranking (national/international levels).

Referees in basketball are required to coordinate their DM, while making accurate decisions in fast-paced game situations applying anticipation and immediate dynamic adjustments (Hossner et al., 2019; MacMahon et al., 2015). In this respect, the use of shared mental models which refer to the knowledge structures that team members possess, could help referees to predict subsequent situations and coordinate their actions accordingly. When different team members perceive a game situation in a similar manner, these knowledge structures could help them improve their DM processes (Cannon-Bowers and Salas, 2001).

Team trust is another important officiating variable that characterizes effectively the functioning of shared mental models. This is true especially in game situations where referees are required to coordinate their actions, with no time to communicate or ability to control their environment (Raue et al., 2021; Rico et al., 2008). Shared mental models and team trust greatly depend on the team's collective experiences (i.e., the longevity of the team), that enable them to develop team-related knowledge. Such knowledge may refer to abilities, behavioural tendencies, roles, and skills (Blickensderfer et al., 2010). We therefore propose that shared team experiences and their effect on performance should be the focus of future studies on referees' DM in dynamic team sports such as basketball.

As for the distance between the position of the referee and the actual officiating situation, our results revealed that national level referees were standing at a similar distance from the officiating situation when the decision was right or wrong. The same is true for the international level referees; however, they tended to be positioned further away from the event than their national level counterparts. This finding is in line with De Oliveira et al. (2011), who did not find a significant association between referees' distance from a foul play and the accuracy of their DM. In other words, distance was not found to be the most important factor in officiating accuracy.

It is evident that when applying the 3PO principle in basketball, all three referees oversee the game. However, only one of the three has primary coverage (namely, the responsibility area) at any given point in the game. It seems that maintaining a position outside the primary coverage area had the potential to hinder the referee's DM accuracy. In a previous study, Wang and Hsieh (2016) analysed performances of basketball referee teams and found that teamwork (or as they labelled it – *teamwork capability*), was associated with the referees' game performance, so that when the referees worked together well, officiating performances were better. Therefore, when applying the 3PO principle, the instructional challenge seems to be to find the most appropriate preparation/training program aimed at improving teamwork among the three referees.

Indeed, observing the actual positions of the referees and their distance from the given officiating situation in comparison to their position derived from the 3PO principle, could assist in evaluating DM accuracy among basketball referees. However, additional studies are required to examine why some officiating decisions are made when standing outside their area of responsibility. It is particularly interesting to investigate why the more experienced, top-level referees make calls when they are standing far away from the officiating situation – actually, in an incorrect position (according to the 3PO principle).

## Limitations and Future Research

This study is exploratory. As such, the results should be further validated in other basketball settings such as in highly professional contests (e.g., the NBA and EuroLeague). In order to strengthen the use of the present methodology in studies on basketball officiating, additional variables should be addressed, such as the referees' gaze behaviour (e.g., Brams et al., 2019). Measuring gaze behaviour in sport settings could increase our understanding of how performers relate to relevant environmental cues. In a recent review on gaze behaviour in sports' referees, data were discussed from soccer, softball, ice hockey, rugby, and team handball, yet not basketball (Ziv et al., 2022). Therefore, basketball laboratory-simulated conditions should be developed in order to enable the researcher not only to collect gaze data, but also to analyse verbal reports provided by the referees. Such collected reports could lead to better understanding of why referees make calls in game situations without precisely following the 3PO Principle.

### Practical Implications for Preparation/Training Programs for Referees in Basketball

A number of practical implications can be derived from our findings. First, teams of basketball referees may benefit from the composition of referees from both the national and the international level, as the latter could upgrade DM of the former. Moreover, preparation/training programs for referees in basketball should place an emphasis on improving the internal hierarchy and team coordination among the three referees in line with the structure of the 3PO principle. A focus should be put on preventing an overlap between the three referees, as well as on enabling each referee to make decisions within his responsibility area. Therefore, referees should be aware of their making calls from (in)correct positions while adopting the 3PO principle, which should of course improve their DM accuracy. Referees should be encouraged not to respond when they are outside their responsibility area, thereby adopting a more cautious approach which allows their colleague/s to make the call when needed.
